# Unusual and unique distribution of anal high-risk human papillomavirus (HR-HPV) among men who have sex with men living in the Central African Republic

**DOI:** 10.1371/journal.pone.0197845

**Published:** 2018-05-24

**Authors:** Ralph-Sydney Mboumba Bouassa, Marcel Mbeko Simaleko, Serge Police Camengo, Christian Diamant Mossoro-Kpinde, David Veyer, Mathieu Matta, Leman Robin, Jean De Dieu Longo, Gérard Grésenguet, Hélène Péré, Jean-François Meye, Laurent Belec

**Affiliations:** 1 Ecole Doctorale Régionale d’Infectiologie Tropicale de Franceville, Franceville, Gabon; 2 Ecole Doctorale Bio Sorbonne Paris Cité, BioSPC, Paris Descartes, Paris, France; 3 Centre National de Référence des Infection Sexuellement Transmissibles et de la Thérapie Antirétrovirale, Bangui, Central African Republic; 4 Servivce de Gastro-entérologie, Hôpital de l’Amitié, Bangui, Central African Republic; 5 Faculté des Sciences de la Santé de Bangui, Bangui, Central African Republic; 6 Laboratoire National de Biologie Clinique et de Santé Publique, Bangui, Central African Republic; 7 Laboratoire de virologie, Hôpital Européen Georges Pompidou, Paris, France; 8 Université Paris Descartes, Sorbonne Paris Cité, Paris, France; 9 Unité de Recherches et d’Intervention sur les Maladies Sexuellement Transmissibles et le SIDA, Département de Santé Publique, Faculté des Sciences de la Santé de Bangui, Bangui, Central African Republic; 10 Service de Gynécologie Obstétrique, Centre Hospitalo-Universitaire d’Angondjé, Libreville et Faculté de Médecine de Libreville, Université des Sciences de la Santé, Libreville, Gabon; Laboratoire National de Santé, LUXEMBOURG

## Abstract

**Background:**

High-risk (HR) human papillomavirus (HPV) infection remains a great concern in relation to African men who have sex with men (MSM), especially those infected with HIV. The prevalence of HR-HPV and associated risk factors was estimated in a cross-sectional observational study covering MSM living in Bangui, Central African Republic.

**Methods:**

MSM receiving care at the *Centre National de Référence des Infections Sexuellement Transmissibles et de la Thérapie Antirétrovirale*, Bangui, were included. HIV serostatus and socio-demographic and behavioral characteristics were collected. HPV DNA was detected and genotyped on anal swabs using Anyplex™ II HPV28 test (Seegene, South Korea), and HSV DNA by in-house real-time PCR. Logistic regression analyses were used to determine risk factors associated with HPV outcomes.

**Results:**

42 MSM (mean age, 23.2 years; range, 14–39) including 69.1% HIV-1-positive and 30.9% HIV-negative were prospectively enrolled. The prevalence of anal HPV was 69.1%, including 82.7% of HR-HPV which were multiple in 52.0%. The most prevalent genotypes were HPV-35, HPV-58, HPV-59 and HPV-31. While, HPV-16 and HPV-18 were present in a minority of samples. Multiple HR-HPV infection was more frequent in HIV-positive MSM (41.4%) with 2.7 genotypes per anal samples than in HIV-negative (7.7%) with 1.5 genotypes per anal samples. HPV types included in the prophylactic Gardasil-9^®^ vaccine were detected in 68.9% of specimens and HPV-58 was the most frequently detected. MSM infected by HPV-16 and HPV-18 were all infected by HIV-1. Few anal swabs (11.9%) contained HSV-2 DNA without relationship with HPV detection. Condomless receptive anal intercourse was the main risk factor to being infected with any type of HPV and condomless insertive anal intercourse was significantly less associated with HPV contamination than receptive anal intercourse (Odd ratio = 0.02).

**Conclusion:**

MSM in Bangui are at-risk of HIV and HR-HPV anal infections. The unusual distribution of HPV-35 as predominant HPV suggests possible geographic specificities in the molecular epidemiology of HR-HPV in sub-Saharan Africa. Scaling up prevention strategies against HPV infection and related cancers adapted for MSM in Africa should be prioritized. Innovative interventions should be conceived for the MSM population living in Bangui.

## Introduction

Men who have sex with men (MSM) in sub-Saharan Africa constitute a core group for several sexual transmitted infections (STI), including human immunodeficiency virus (HIV) [1–4], human papillomavirus (HPV) and herpes simplex virus type 2 (HSV-2) infections [5–8]. HPV infection is the most common viral STI in the world and high-risk oncogenic (HR)-HPV genotypes are responsible for 7.7% of all cancers in developing countries [9,10]. Therefore, anal HR-HPV infection is a steady increasing health problem for MSM because it causes anal cancer. Indeed, MSM are about 20 times more likely susceptible than heterosexual men to develop HPV-related anal cancer and HIV-infected MSM are at an even greater risk [11,12]. Indeed, HIV infection is considered as an independent risk factor strongly associated with increased risk for acquiring HR-HPV anal infection [13–15]. *In vitro* interactions between HSV-2 and HPV suggest that HSV-2 infection could constitute a cofactor of anal carriage for HPV infection [16], especially in the epidemiological context of sub-Saharan Africa, where HSV-2 infection is highly prevalent [17,18], and constitutes the first cause of genital ulcer [17–19].

The burden of HPV infection in MSM living in sub-Saharan Africa has nonetheless been poorly documented [8].However, recent reports from South Africa [6] and Nigeria [7] emphasize very high prevalences of anal HR-HPV infection, ranging from 57.6 to 70.1%, in MSM living in sub-Saharan Africa, especially in those co-infected with HIV [6,7]. The reported prevalences of anal HR-HPV DNA among MSM living in sub-Saharan Africa appear higher than those usually recorded in studies conducted in developed countries, which range from 20.9% to 65% [20–23]. Anal HR-HPV in African MSM was strongly associated with high-risk sexual behaviors such as having sex with men only, engaging in group sex and practicing condomeless receptive anal intercourse [6,7]. Interestingly, a wide diversity of predominant HPV genotypes was observed in South African and Nigerian studies, suggesting the possibility of unique spatial distributions of HPV diversity by regions within sub-Saharan Africa [6,7]. Thus, Müller et al. described in South Africa a distribution quite similar to that commonly observed throughout the world with HPV-16 as predominant genotype [6]. In contrast, Nowak et al. depicted in Nigeria an atypical distribution profile with the non-vaccine HR-HPV-35 as the predominant genotype circulating in MSM [7].Although limited, these observations highlight that MSM in sub-Saharan Africa constitute a high-risk core group for HR-HPV infection and that the distribution of the main HPV genotypes involved in anal cancers in African MSM could be relatively different from that generally observed. Finally, in order to implement effective HPV vaccine-based prevention adapted to every sub-Saharan African region, it is important to establish the molecular distribution of predominant HR-HPV genotypes circulating in African MSM.

Little data is available on MSM living in the Central African Republic (CAR). One recent preliminary serosurvey conducted on MSM in Bangui highlighted that MSM are an identifiable core group accumulating high-risk sexual behaviors for STI, and high prevalence of HIV (25%), hepatitis B (17%) and syphilis (4%) [24].Herein, we designed a cross-sectional study to assess the prevalence and type distribution of anal HPV infection and associated risk factors, including sexual behavior and HSV-2 infection, in a population of HIV-infected and HIV-uninfected MSM living in Bangui, the capital city of the CAR.

## Material and methods

### Study population, medical interventions and data collection

The Centre National de Référence des Infections Sexuellement Transmissibles et de la Thérapie Antirétrovirale (CNRIST/TAR) of Bangui includes both care for general population and specific care towards the MSM population of Bangui. MSM regularly attend the STI clinic for HIV and STI screening and care, to receive specific treatment, HIV counseling and for those positive, for HIV global support. For purposes of the study, a specific strategy involving peer educators was adopted in order to confirm the accuracy of homosexuality of the included MSM attending the CNRIST/TAR. Thus, inclusion criteria were to be in majority age (age ≥18 years), to be approved as having sex with men by his peers, to get possible follow up for at least 3 months, and to have a fully informed medical and socio-demographic record. At inclusion, a standardized interview was conducted to collect socio-demographic characteristics and behavioral data, including age, ethnic group, number of sexual male partners in the last 6 months, sexual orientation, frequency of condom use, sexual practices, HIV status and antiretroviral treatment (ART) for those already aware of their positive HIV status and finally to advise participants about HIV and associated STIs.

After the interviews, MSM undertook medical appointments including clinical examinations and biological investigations for the diagnosis of the most common STIs including HIV (for those who did not know their HIV-serological status), syphilis and hepatitis B. Biological results were returned 72 hours after and those positive for STIs received adapted treatment. HIV-positive MSM were enrolled in the HIV cohort followed in the CNRIST/TAR. The medical intervention package consisted of HIV/STI counseling, condom distribution, clinical examination, biological monitoring, and medical care for patients infected with STIs and HIV. A Medical professional carried out physical and clinical examinations to check patients for symptoms of potential diseases. For HIV/STI counseling and condom distribution, a 7 to 10-minute interactive conversation on HIV, STIs, their modes of transmission and effective prevention mean with a special emphasis on condom use as an easy and effective prevention tool, was carried out by health care assistants. At the end, the health care assistants distributed as many condoms as required to the participants.

### Samples and processing

Plasma or serum samples from blood collected by venipuncture in each MSM were used for serological testing of HIV infection, as recommended by national guidelines [25]. HIV-positive status was determined by the national serological testing algorithm using Genscreen ULTRA Combo HIV Ag/Ab test (Bio-Rad Laboratories, Washington, USA), an enzyme immunoassay kit for the simultaneous detection of HIV p24 antigen and antibodies to HV-1 (group M and O) and HIV-2 in serum or plasma. The positive tests were confirmed by an enzyme-linked immunosorbent assay [ELISA] (Vironostika® HIV Uni-Form II Ag/Ab, bioMérieux Marcy l'Etoile, France). Specimens for molecular testing were obtained by inserting a moistened polyester swab into the anal canal, rotating 5 times and then removing. The swab was immediately placed into a sampler tube and frozen at -80°C before the DNA extraction procedure.

### HPV detection and genotyping

DNA was extracted from the anal swab specimen using the DNeasyBlood and Tissue kit, as recommended by the manufacturer (Qiagen, Hilden, Germany). The detection of HPV DNA and the distribution of genotypes were done using Anyplex™ II HPV28 detection test (Seegene, Seoul, South Korea). Anyplex™ II HPV28 detection test was performed as recommended by the manufacturer with 5μl of DNA in each of the two reaction mixtures (20 μl) with the primers A and B [26]. According to the International Agency for Research on Cancer (IARC) nomenclature [27], Anyplex™ II HPV28 detection test distinguishes 28 HPV genotypes, including 13 high-risk types (HR-HPV -16, -18, -31, -33, -35, -39, -45, -51, -52, -56, -58, -59, and -68), 9 low-risk (LR) types (LR-HPV -6, -11, -40, -42, -43, -44,-53, -54 and -70) and then, 6 genotypes reported as possibly carcinogenic (HPV-26, -61, -66, -69, -73 and -82). The process is carried out in 2 reactions by taking advantage of the 5 dyes that can be resolved on the CFX96™ real-time PCR instrument (Bio-Rad, Marnes-la-Coquette, France) [26]. Anyplex™ II HPV28 has been evaluated for several years and is as suitable for HPV genotyping as other molecular assays commonly used for genotyping [26, 28–32]. Data recording and interpretation were automated with Seegene viewer software (Seegene, Seoul, South Korea), according to the manufacturer’s instructions. A swab sample was considered positive for any HPV if containing any of the 28 types included in the Anyplex™ II HPV28 detection test; positive for multiple HPV when containing at least 2 types of the 28 HPV types included in genotypic test; HR-HPV positive and multiple HR-HPV positive when containing respectively at least 1 HR-HPV type and at least 2 HR types of the 19 HR types of the Anyplex™ II HPV28 detection test, irrespective of the presence of LR-HPV.

### HSV detection in the anal tract

HSV DNA was detected from 1 μg of DNA extracted from the anal swab specimen by in-house real-time PCR using LightCycler® 480 Real-Time PCR (Roche molecular diagnostics, California, USA), as previously described [33,34]. HSV genotypes (HSV-2 versus HSV type 1) were assessed by the final melting curve, [33,34].

### Statistical analyses

Statistical analyses were conducted using IBM^®^ SPSS^®^ Statistics 20 software (IBM, SPSS Inc, Armonk, New York, USA). P-values were calculated using Pearson’s χ^2^ test or Fisher's exact test for categorical variables and the non-parametric Mann-Whitney *U*-test for quantitative variables. Logistic regression models were assessed to evaluate the association of each independent variable [i.e., age at enrollment, HIV-1 infection, sex of sexual partner (MSM-exclusively or MSMW), the number of sexual male partners in the last 6 months, sexual practices in the last 6 months (condomless receptive anal sex; condomless insertive anal sex; regular receptive oral sex) and the HSV-2 DNA detection in anal swab] with the HPV type-specific anal infections (i.e., anal infection by any type of HPV, multiple types of HPV, HR-HPV and multiple HR-HPV). All variables statistically significant (P < 0.05) in univariate analyses were entered into multivariate logistic regression models. Crude Odds ratio (cOR) and adjusted Odds ratio (aOR) were calculated, as appropriate along with 95% confidence intervals (CI). For variable giving infinite OR, the Odds ratios and their confidence intervals were recalculated, using the statistical software package R (available at https://www.r-project.org/) and the hypothesis test inversion method, as previously described [35]. The final multivariate model for any HPV outcome included condomless receptive and insertive anal sex and regular receptive oral sex. For multiple HPV outcome, the final multivariate model included condomless receptive and insertive anal sex and regular receptive oral sex. For HR-HPV outcomes, the final multivariate model included condomless insertive anal sex and regular receptive oral sex. Finally, for the multiple HR-HPV outcomes variable, the final multivariate model included HIV infection and condomless insertive anal sex.

### Ethics statement

The study was formally approved by the Scientific Committee Faculty of Health Sciences of Bangui (“Comité Scientifique Chargé de la Validation des Protocoles d’Etudes et des Résultats”/”CSCVPER”) (agreement UB/FACSS/CSCVPER), which constitutes the National Ethical Committee. All MSM participants were of majority age and gave their informed oral consent to participate in the study. For each MSM, the record of the consent was documented in each questionnaire. This consent procedure was formally approved by the National Ethical Committee.

## Results

### Characteristics of study population

Forty-two participants were included and their socio-demographic, sexual behavior, clinic-biological characteristics are shown in the Table 1. Among them, 29 (69.1%) were infected by HIV-1 whereas 13 (30.9%) were HIV-negative.

**Table 1:** Baseline characteristics according to sexual behavior and HIV serostatus among the 42 study men who have sex with men (MSM) living in Bangui, Central African Republic.

	MSM	MSM-exclusively	MSMW	P^*^	HIV+	HIV-	P^*^
Number of patients	42	8	34		29	13	
**Age**							
All age [mean (SD), years]	23.2 (5.6)	20.5 (0.7)	23.2 (4.9)	NS	19.5 (4.9)	21.5 (5.6)	NS
18–19	11 (26.2)	2 (25) [0.0–55.1]	9 (26.5) [11.6–41.3]	NS	5 (17.3) [3.5–30.9]	6 (46.2) [19.1–73.3]	NS
20–29	23 (54.7)	5 (62.5) [28.9–96.1]	18 (64.7) [36.2–69.7]		18 (62.1) [44.4–79.7]	5 (38.5) [12.1–64.9]	
≥30	8 (19.1)	1 (12.5) [0.0–35.4]	7 (8.8) [7.0–34.2]		6 (20.7) [5.9–35.4]	2 (15.4) [0.0–35.0]	
**HIV-1 infection** [n (%)]	29 (69)	8 (100) [100–100]	21 (61.7) [45.4–78.1]	0.04	NA	NA	NA
**ART at inclusion** [n (%)]	10 (23.8)	4 (50) [15.4–84.6]	6 (17.4) [4.8–30.5]	0.07	10 (34.5)	NA	NA
**CD4 T cell counts** [n (%),cells/μl]							
> 500	13 (30.9)	4 (50.0) [15.4–84.6]	9 (42.8) [21.7–64.1]	NS	13 (44.8)	NA	NA
351–500	13 (30.9)	3 (37.5) [3.9–71.1]	10 (47.6) [26.3–68.9]		13 (44.8)	NA	
≤ 350	3 (7.2)	1 (12.5) [0.0–35.4]	2 (9.5) [0.0–22.1]		3 (10.4)	NA	
**Sex of sexual partners over the last 6 months** [n (%)]							
MSM-exclusively	8 (19.1)	NA	NA	NA	8 (27.6) [11.3–43.8]	0 (0) [0.0–0.0]	0.04
MSMW	34 (80.9)	NA	NA		21 (72.4) [56.2–88.7]	13 (100) [100–100]	
**Numbers of sexual male partners in last 6 months** [n (%)]							
1–5	40 (95.3)	8 (100) [100–100]	32 (94.1) [86.2–100]	NS	28 (96.5) [89.9–100]	12 (92.3) [77.8–100]	NS
> 5	2 (4.7)	0 (0) [0.0–0.0]	2 (5.9) [0.0–13.8]		1 (3.5) [0.0–10.1]	1 (7.7) [0.0–22.2]	
**Sexual practices in last 6 months** [n (%)]							
Condomless receptive anal sex	30 (71.4)	7 (87.5) [64.6–100]	23 (67.6) [51.9–83.4]	NS	22 (75.8) [60.3–91.4]	8 (61.5) [35.1–87.9]	NS
Condomless insertive anal sex	15 (35.7)	1 (12.5) [[0.0–35.4]	14 (41.2) [24.6–57.7]	NS	10 (34.5) [17.18–51.78]	5 (38.5) [12.1–64.9]	NS
Regular receptive oral sex	28 (66.6)	8 (100) [100–100]	20 (58.8) [42.3–75.4]	0.03	25 (86.2) [73.6–98.7]	3 (23.1) [0.2–45.9]	0.0001
**HSV-2 DNA in swab** [n (%)]	5 (11.9)	1 (12.5) [0.0–35.4]	4 (11.7) [0.9–22.6]	NS	5 (17.3) [3.5–30.9]	0 (00) [0.0–0.0]	NS
**HPV DNA detection and types** [n (%)]							
HPV DNA in swab	29 (69.1)	7 (87.5) [64.6–100.0]	22 (64.7) [48.6–80.7]	NS	21 (72.4) [56.15–88.68]	8 (61.5) [35.1–87.9]	NS
Multiple types of any HPV	25 (59.5)	7 (87.5) [64.6–100.0]	18 (52.9) [36.2–69.7]	NS	19 (65.5) [48.22–82.82]	6 (46.2) [19.1–73.3]	NS
LR-HPV	20 (47.6)	5 (62.5) [28.9–96.1]	15 (44.1) [27.4–60.8]	NS	14 (48.2) [30.1–66.4]	6 (46.2) [19.1–73.3]	NS
HR-HPV	24 (57.1)	5 (62.5) [28.9–96.1]	19 (55.8) [39.2–72.6]	NS	18 (62.1) [44.4–79.7]	6 (46.2) [19.1–73.3]	NS
Multiple types of HR-HPV	13 (30.9)	3 (37.5) [3.9–71.1]	10 (29.4) [14.1–44.7]	NS	12 (41.4) [23.5–59.3]	1 (7.7) [0.0–22.2]	0.007
HPV-16	4 (9.5)	2 (25.0) [0.0–55.1]	2 (5.8) [0.0–13.8]	NS	4 (13.8) [1.2–26.3]	0 (0) [0.0–0.0]	NS
HPV-18	3 (7.2)	1 (12.5) [0.0–35.4]	2 (5.8) [0.0–13.8]	NS	3 (10.3) [0.0–21.4]	0 (0) [0.0–0.0]	NS
HPV-16 and HPV-18	1 (2.3)	1 (12.5) [0.0–35.4]	0 (0) [0.0–0.0]	NS	1 (3.4) [0.0–10.1]	0 (0) [0.0–0.0]	NS
Any 4-valent vaccine types**	11 (26.2)	4 (50.0) [15.4–84.6]	7 (20.6) [7.0–34.2]	NS	9 (31.1) [14.2–47.8]	0 (0) [0.0–0.0]	0.04
Multiple 4-valent vaccine types	1 (2.3)	1 (12.5) [0.0–35.4]	0 (0) [0.0–0.0]	NS	1 (3.5) [0.0–10.1]	0 (0) [0.0–0.0]	NS
Any 9-valent vaccine types***	20 (47.6)	4 (50.0) [15.4–84.6]	16 (47.1) [30.3–63.8]	NS	15 (51.7) [33.6–69.9]	5 (38.5) [12.2–64.9]	NS
Multiple 9-valent vaccine types	9 (21.5)	2 (25.0) [0.0–55.1]	7 (20.6) [7.0–34.2]	NS	7 (24.2) [8.5–39.7]	2 (15.4) [0.0–35.0]	NS

* P-value calculated using Pearson’s χ2 test or Fisher's exact test for categorical variables and the non-parametric Mann-Whitney U-test for non-categorical variables.

** The 4-valent Gardasil-4® vaccine (Merck & Co. Inc., New Jersey, USA) is effective against HPV genotypes 6, 11, 16 and 18;

*** The 9-valent Gardasil-9® vaccine is effective against HPV genotypes 6, 11, 16, 18, 31, 33, 45, 52 and 58.

ART: Antiretroviral treatment; HIV-1: Human immunodeficiency virus-1; HSV-2: Herpes simplex virus-2; HR-HPV: high-risk human papillomavirus; LR-HPV: low-risk human papillomavirus; MSM: men who have sex with men; MSM-exclusively: men who have sex only with men; MSMW: men who have sex with both men and women; NA: Not applicable; NS: Not significant.

Overall, the study population was exclusively constituted by black native people and comprised mainly young men (mean age: 23.2 years; range, 18–39). All were living in 4 (out of 10) districts of the capital city Bangui which were neighboring. The majority of participants (34/42; 80.9%) reported having sex in the last 6 months with both men and women (MSMW), whereas a minority (8/42; 19.1%) reported having sex in the last 6 months exclusively with men (MSM-exclusively).The prevalence of HIV-1 infection was higher in MSM-exclusively (8/8; 100%; 95%CI: 100–100%) than in MSMW (21/34; 61.7%; 95%CI: 45.4–78.1%) (P = 0.04). At inclusion, only 10 of 29 (34.5%) HIV-infected MSM, including 4 MSM-exclusively and 6 MSMW, were tacking antiretroviral treatment (ART) according to the 2015-World Health Organization (WHO) consolidated guidelines [36]. Among them, only 6, including 4 MSM-exclusively and 2 MSMW, showed a baseline CD4 cell count above 500 cells/μl. Most (40/42; 95.3%) of the MSM reported having sexual intercourse with at least 1 to 5 partners (median: 4; range, 1–5) in the last 6 months and most of them (30/42; 71.4%) had condomless receptive anal sex during the past 6 months. The group of MSM-exclusively reported having 7-time more receptive anal sex than insertive anal sex [receptive anal sex: 7/8 (87.5%), 95%CI: 64.6–100.0%; insertive anal sex: 1/8 (12.5%), 95%CI: 0.0–35.4%]. Furthermore, MSM-exclusively reported more likely to have receptive oral sex than MSMW [MSM-exclusively: 8/8 (100.0%), 95%CI: 100.0–100.0%; MSMW: 20/34 (58.8%), 95%CI: 42.28–75.37%; P<0.03]. Lastly, HIV-infected MSM reported practicing more regularly receptive oral sex than HIV-negative MSM [25/29 (86.2%); 95%CI: 73.6–98.7% *versus* 3/13 (23.1%); 95%CI: 0.2–45.9%; P<0.001].

Finally, the study MSM were generally free of clinical STI symptoms at admission. Thus, only 5 MSM showed herpetic genital recurrences (n = 2), syphilis genital ulcer (n = 1), anal warts (n = 1) and anal infection due to Neisseria gonorrhea (n = 1). The seroprevalences of syphilis and hepatitis B (HBs antigen) at inclusion were 12% and 14%, respectively.

### HSV DNA detection

Only 5 (11.9%) of the study MSM were positive for HSV-2 in anal samples (1 MSM-exclusively and 4 MSMW); all of them were coinfected with HIV-1 and 4 of them showed multiple anal HR-HPV infections.

### HPV prevalence and genotype distribution

**Fig 1:**
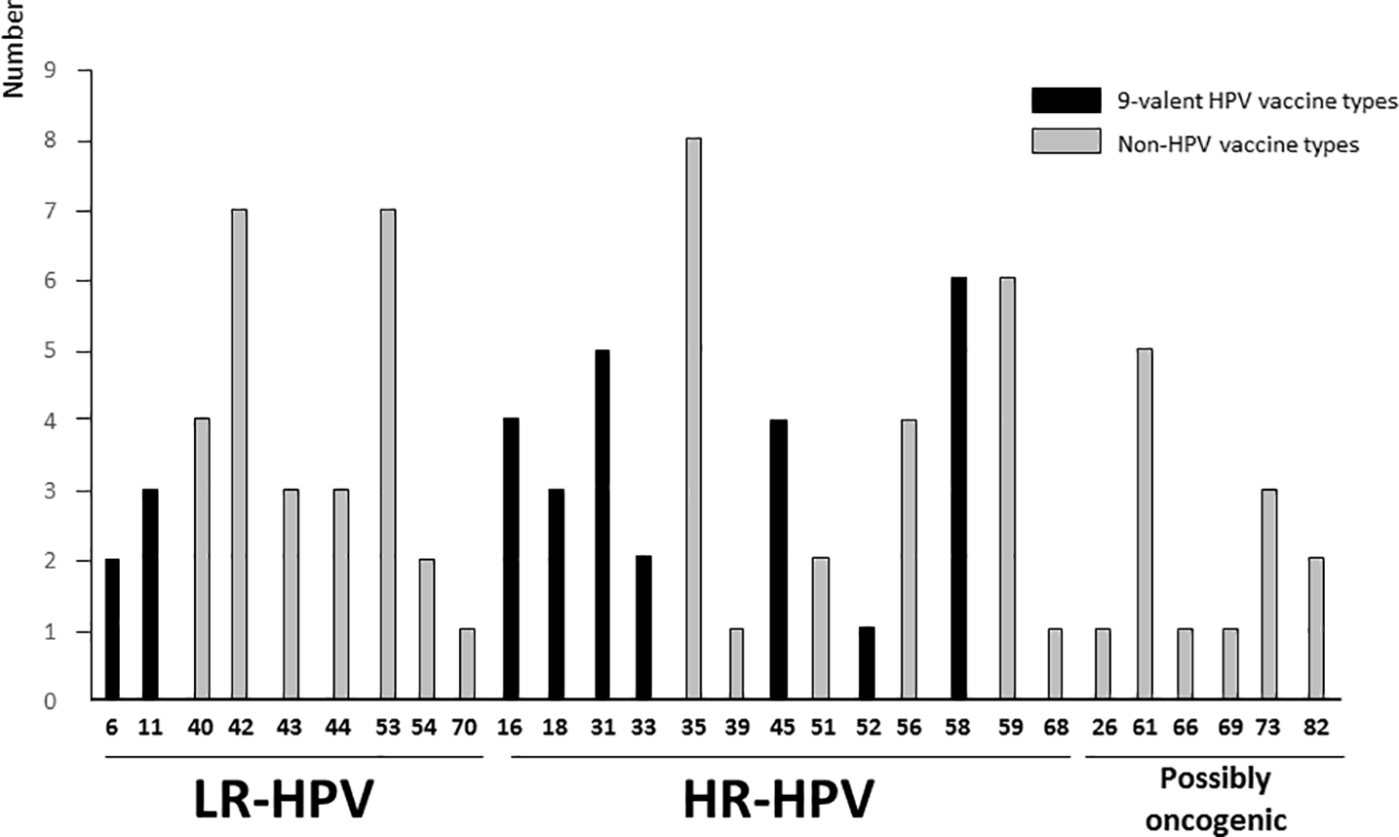
Distribution of anal HPV types included (or not included) in the 9-valent Gardasil-9® vaccine. Number of low-risk (LR) and high-risk (HR) HPV genotypes in 29 anal swabs positive for HPV DNA by molecular biology according to their possible prevention by 9-valent HPV vaccine among study men who have sex with men (n = 42) living in the Central African Republic. Nota bene. The 9-valent Gardasil-9® vaccine (Merck & Co. Inc., New Jersey, USA) is effective against HPV genotypes 6, 11, 16, 18, 31, 33, 45, 52 and 58.

As shown in Table 1, the overall HPV prevalence in study population was 69.1% (29/42) with 82.7% (24/29) of HR-HPV DNA-positive samples. Most (25/42; 59.5%) anal swabs contained multiple HPV genotypes and 52.0% (13/25) of them contained an average of 2.7 HR-HPV (range, 1 to 4) per anal swab sample. About 24 out of 29 (82.7%; 95%CI: 69.01–96.51%) HPV-positive anal specimens showed at least 1 HR-HPV and only 37.9% (11/29) showed single HR-HPV infections. The distribution of HPV genotypes in HPV DNA-positive anal samples is depicted in Fig 1.

HR-HPV-35 was the predominant genotype (8/29; 27.6%), followed by LR-HPV types 42 and 53 with a prevalence of 24.1% (7/29), HR-HPV types 58 and 59 with 20.7% (6/29) and HR-HPV types 31 and 61 with 17.2% (5/29). HPV-16 and HPV-18 were found in a minority of HPV-positive swabs [4/29 (13.8%) and 3/29 (10.3%), respectively] (Fig 1).

**Fig 2:**
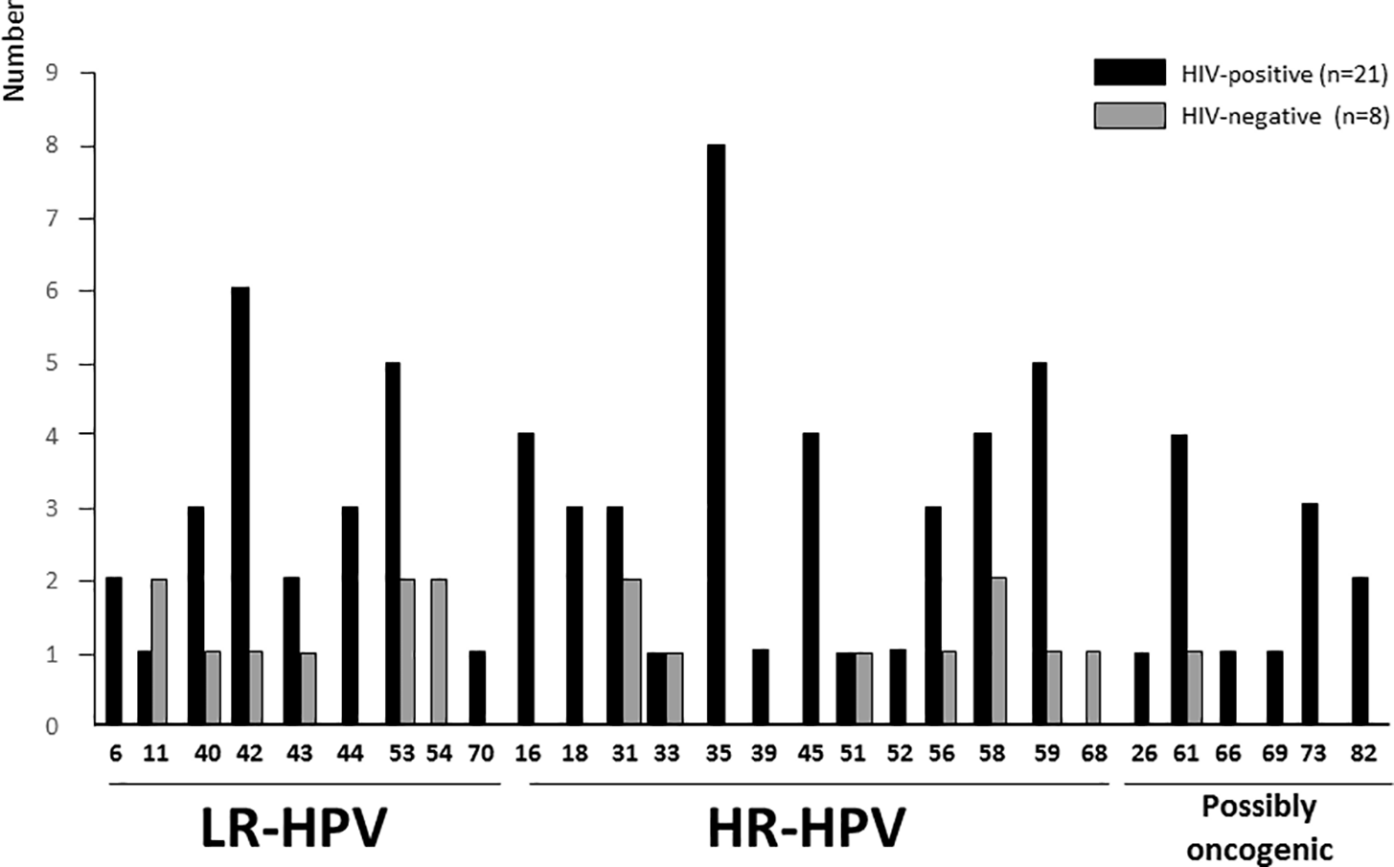
Distribution of anal HPV types according to the HIV-serostatus. Number of low-risk (LR) and high-risk (HR) HPV genotypes in 29 anal swab positive for HPV DNA by molecular biology according to HIV-serostatus among study men who have sex with men (n = 42) living in the Central African Republic.

Among the 21 HIV-1-infected MSM with anal HPV infection, 19 (90.5%) had multiple HPV and 20 (95.3%) were co-infected with HR-HPV (Fig 2 and Table 1). Multiple anal HR-HPV types were detected more frequently in HIV-1-infected than HIV-negative MSM (41.4% versus 7.7%; P<0.01). The mean number of HR-HPV genotypes more frequently detected in HR-HPV-positive anal swabs was 2.7 (range 1–4) in HIV-positive MSM and 1.5 (range 1–4) in HIV-negative MSM (P = 0.07). MSM positive for HPV-16, HPV-18 and HPV-35 were all HIV-1-infected (Fig 2) and only one sample was simultaneously infected with both HPV-16 and HPV-18.

Possible efficiencies of anal HPV prevention by 4- and 9- valent Gardasil® vaccines were further assessed. 37.9% (11/29) of the HPV-positive anal swabs were infected with at least 1 of the 4 genotypes covered by the Gardasil-4 vaccine. Regarding the Gardasil-9 vaccine, 68.9% (20/29) of HPV-positive anal samples contained at least 1 HPV type included in the 9-valent HPV vaccine and 45% (9/20) of them contained multiple HPV genotypes (Fig 2 and Table 1). The predominant HR-HPV genotype included in the 9-valent HPV vaccine detected in study anal swabs was the type 58 with a prevalence of 20.7% (20/29) (Fig 2).

Risk factors associated with HPV infection. The associations between anal HPV infection, including anal infection by any type of HPV, multiple types of HPV, and HR-HPV type and multiple types HR-HPV, with their potential risk factors were assessed by logistic regression analysis, as shown in Table 2.

**Table 2:** Univariate and multivariate logistic regression analyses for HPV-associated risk factors 42 study men who have sex with men (MSM) living in Bangui, Central African Republic.

	Any HPV					Multiple HPV					HR-HPV					Multiple HR-HPV				
Number of patients	29					25					24					13				
Risk factors	n (%)	cOR (95%CI)	P*	aOR (95%CI)	P*	n (%)	cOR (95%CI)	P*	aOR (95%CI)	P*	n (%)	cOR (95%CI)	P*	aOR (95%CI)	P*	n (%)	cOR (95%CI)	P*	aOR (95%CI)	P*
**Age** (years)																				
18–19	8 (27.6)	0.7 (0.16–3.5)	0.9	NA**	NA	8 (32)	2.2 (0.5–9.9)	0.3	NA	NA	5 (19.2)	0.4 (0.1–1.6)	0.4	NA	NA	2 (12.5)	0.9 (0.2–3.8)	0.8	NA	NA
20–29	14 (48.2)	1.13 (0.1–15.5)		NA	NA	12 (48)	0.9 (0.3–3.5)		NA	NA	13 (50.0)	1.8 (0.5–6.4)		NA	NA	8 (50.0)	1.4 (0.4–5.1)		NA	NA
≥ 30	7 (24.1)	1.7 (0.1–20.3)		NA	NA	5 (20)	0.2 (0.0–2.1)		NA	NA	6 (23.5)	1.9 (0.2–20.6)		NA	NA	3 (18.7)	0.5 (0.05–5.4)		NA	NA
**HIV-1 infection**	21 (74.4)	2.25 (0.6–8.7)	0.2	NA	NA	19 (76)	3.16 (0.3–27.6)	0.3	NA	NA	20 (76.9)	3.3 (0.9–12.7)	0.07	NA	NA	15 (93.7)	15.0 (1.4–161.1)	0.003	5.2 (0.4–70.9)	0.2
**Sex of sexual partners**																				
**MSM-exclusively**	7 (24.2)	3.8 (0.4–34.8)	0.2	NA	NA	7 (28)	6.2 (0.7–56.2)	0.08	NA	NA	6 (23.1)	0.6 (0.04–7.8)	0.6	NA	NA	4 (25)	1.33 (0.2–9.1)	0.7	NA	NA
**MSMW**	22 (75.8)	0.3 (0.03–2.4)	0.2	NA	NA	18 (72)	0.2 (0.0–1.5)	0.08	NA	NA	20 (76.9)	1.6 (0.12–21.7)	0.6	NA	NA	12 (75)	0.75 (0.11–5.1)	0.7	NA	NA
**Numbers of sexual male partners in last 6 months**																				
1–5	27 (93.1)	0.0 (0.0–100.0)	0.9	NA	NA	23 (92)	0.0 (0.0–100.0)	0.9	NA	NA	24 (92.3)	0.0 (0.0–100.0)	0.4	NA	NA	14 (87.5)	0.0 (0.0–100.0)	0.9	NA	NA
> 5	2 (6.8)	0.0 (0.0–100.0)		NA	NA	2 (8)	0.0 (0.0–100.0)		NA	NA	2 (7.7)	0.0 (0.0–100.0)		NA	NA	2 (12.5)	0.0 (0.0–100.0)		NA	NA
**Sexual practices in last 6 months**																				
**Condomless insertive anal sex**	4 (13.8)	0.01 (0.002–0.13)	< 0.01	0.02 (0.001–0.18)	< 0.01	3 (12)	0.4 (0.03–5.3)	< 0.05	0.05 (0.01–0.3)	0.01	3 (11.5)	0.03 (0.0–0.2)	<0.001	0.022 (0.0–0.24)	0.002	2 (12.5)	0.1 (0.02–0.65)	0.008	0.2 (0.02–1.1)	0.05
**Condomless receptive anal sex**	29 (100)	14.1 (2.9–68.4)	0.001	4.9 (0.5–47.1)	0.16	25 (100)	5.9 (1.4–24.7)	0.014	1.4 (0.2–11.3)	0.67	24 (100)	5.0 (1.2–20.6)	0.24	NA	NA	13 (100)	1.7 (0.4–7.8)	0.36	NA	NA
**Regular receptive oral sex**	24 (82.7)	11.0 (2.35–49.5)	0.002	10.7 (0.3–118.3)	0.05	21 (84)	7.5 (1.8–31.7)	<0.05	4.8 (0.8–28.2)	0.09	22 (84.6)	16.9 (3.4–83.7)	<0.001	22.3 (2.0–244.1)	0.01	16 (100)	NA	NA	NA	NA
**HSV-2 DNA in swab**	4 (13.8)	1.9 (0.2–19.1)	0.57	NA	NA	4 (16)	3.0 (0.3–30.0)	0.9	NA	NA	4 (15.4)	2.7 (0.3–26.9)	0.4	NA	NA	4 (25)	8.3 (0.8–82.9)	0.06	NA	NA

* *P*-value calculated using Pearson’s χ^2^ test or Fisher's exact test for categorical variables and the non-parametric Mann-Whitney U-test for non-categorical variables

** NA: Not attributable for variables giving crude Odds ratio not significant in univariate analysis (P > 0.05).

ART: Antiretroviral treatment; aOR: adjusted Odds ratio; cOR: crude Odds ratio; HIV-1: Human immunodeficiency virus-1; HSV-2: Herpes simplex virus-2; HR-HPV: high-risk human papillomavirus; LR-HPV: low-risk human papillomavirus; MSM: men who have sex with men; MSM-exclusively: men who have sex only with men; MSMW: men who have sex with both men and women; n: Number (size of study group); NA: Not attributable;CI: Confidence Interval.

No significant differences could be observed between MSM-exclusively and MSMW regarding to the prevalence of HPV DNA detection in the anal samples [7/8 (87.5%) versus 22/34 (64.7%), respectively; P>0.05]. However, the MSM-exclusively group had the highest rates of anal detection of HPV infections, including LR-HPV and HR-HPV with high prevalence of HPV-16 and HPV-18, multiple HPV as well as HPV types included in the 9-valent HPV prophylactic vaccine.

In the univariate analysis, anal infections by any type of HPV and multiple type of HPV were significantly associated with the practice of condomless receptive anal sex (cOR: 14.1, 95%CI: 2.9–68.4%; P = 0.001; cOR: 5.9, 95%CI: 1.4–24.7%; P = 0.014, respectively). Likewise, anal infections by any type of HPV, multiple type of HPV and anal infections with HR-HPV were significantly associated with the practice of receptive oral sex (cOR: 11.0, 95%CI: 2.3–49.5%; P = 0.002; cOR: 7.5, 95%CI: 1.8–31.7%; P<0.05 and cOR: 16.9, 95%CI: 3.4–83.7%; P<0.001, respectively). The anal carriage of multiple anal HR-HPV infection was significantly associated with being infected with HIV (cOR: 15.0, 95%CI: 1.4–161.1%; P = 0.003). Insertive anal sex was significantly associated with a slightly decreased risk of being infected with any HPV type, HR-HPV type and multiple HR-HPV type in the anal canal (cOR: 0.01, 95%CI: 0.002–0.130%; P<0.01; cOR: 0.03, 95%CI: 0.0–0.2%; P<0.001 and cOR: 0.01, 95%CI: 0.02–0.65%; P = 0.008, respectively).

In the multivariate analysis, condomless insertive anal sex and having regular receptive oral sex were the only factors associated with an HPV outcome after adjusting other significant variables in the univariate analysis. Thus, having regular receptive oral sex was a substantial risk factor for being infected with the HR-HPV in the anal canal (aOR: 22.3, 95%CI: 2.0–244.1%; P = 0.01). Likewise, the weak protective effect of practicing insertive anal sex against the acquisition of any HPV type, HR-HPV type and multiple HR-HPV type in the anal canal observed in univariate analysis (cOR: 0.01, 95%CI: 0.002–0.13%; P<0.01; cOR: 0.03, 95%CI: 0.0–0.2%; P<0.001 and cOR: 0.1, 95%CI: 0.02–0.65%; P = 0.008, respectively) was maintained in multivariate analysis (aOR: 0.02, 95%CI: 0.001–0.18%; P<0.01; aOR: 0.022, 95%CI: 0.0–0.24%; P = 0.002 and aOR: 0.20, 95%CI: 0.02–1.1%; P = 0.05, respectively). In addition, the effect of insertive anal sex against the acquisition of multiple type of HPV in the anal canal, which was not significant in univariate analysis (cOR: 0.4, 95%CI: 0.03–5.3%; P<0.05) was upgraded as a weak protective effect in the multivariate analysis (aOR: 0.05, 95%CI: 0.01–0.3%; P = 0.01). In the other hand the strong risk effect of being infected with any HPV and multiple HPV observed in univariate analysis for MSM practicing condomless receptive anal intercourse was downgraded in multivariate analysis until being not significant anymore (aOR: 4.9, 95%CI: 0.5–47.1%; P = 0.16 and aOR: 1.4, 95%CI: 0.2–11.3%; P = 0.669; respectively). Finally, the other explicative variables such as age group, sexual orientation (MSM-exclusively or MSMW), number of sexual male partners and anal HSV-2 infection were not significantly associated with each of the four variables characterizing anal HPV infections that were taken into account in the analysis.

## Discussion

In the present series, the study of MSM living in the Central African Republic and overseen at the *Centre National de Référence des Infections Sexuellement Transmissibles et de la Thérapie Antirétrovirale* of Bangui showed remarkable findings. Firstly, the prevalence of HIV-1 infection in the MSM was notably high (#70%). Secondly, the prevalence of anal HPV was also particularly high (#70%) and unique due to the high prevalence of HR-HPV (82.7%), its high genotypes diversity and the frequent (52%) multiple HR-HPV infections. Thirdly, the distribution of anal HPV in anal samples appeared unusual, the most prevalent genotypes being HPV-35, HPV-58, HPV-59 and HPV-31, while the more classical HPV-16 and HPV-18 were present only in a minority of samples (#25%), likely indicating possible regional clusterization in the diffusion of HR-HPV within the MSM community living in Bangui. Fourthly, HPV types included in the prophylactic Gardasil-9^®^ vaccine were detected in the majority of HPV-positive anal samples (#70%) suggesting that the current 9-valent vaccine could be beneficial for the prevention of HPV-associated disease in this MSM community, although one-third of HPV anal infection would not be prevented. Finally, anal HSV-2 shedding was not associated with anal HPV shedding in our study population, in which the only negative modulatory cofactor for HPV anal carriage was behavioral, the insertive anal intercourse being relatively protective in comparison with receptive anal intercourse. Taken together, our observations indicate for the first time that the MSM community living in Bangui should be at very high-risk for HIV infection as well as HR-HPV anal infections, and strongly suggest that scaling up prevention strategies against HPV infection and related cancers adapted to this highly vulnerable MSM community should be urgently prioritized with innovative interventions.

Very high prevalence of HIV-1 was observed in the study MSM, suggesting that the HIV prevalence in the MSM community of Bangui could be high. This observation is reminiscent to similar reports on MSM living in sub-Saharan Africa. Studies conducted in sub-Saharan African countries in the last 10 years show that HIV prevalence among MSM is more than 5–18 times higher as compared to general population [37, 38]. In Tanzania, the prevalence of HIV in MSM was 17.4% and 3.7% among the general population [3]. Similarly, in Malawi, the prevalence of HIV was 21.4% in MSM and 6.1% in the general population [39]. In Kenya, the HIV prevalences in MSM ranged from 12.3% to 43.0% as compared to 6.1% in the general population [40]. In Ivory Coast, the prevalence of HIV in MSM was as high as 50% as compared to 3.2% in the general population [41].

The prevalence of anal HPV was particularly high (#70%) in this sample of MSM from the Bangui’s community. The high prevalence of anal HR-HPV in our study MSM appeared quite similar to the prevalence reported in MSM living in South Africa and Nigeria, ranging from 58% to 72% [6, 7], but lower than the prevalence reported in young Black American MSM (87%) [42]. Other reports conducted outside Africa showed lower anal HR-HPV prevalence rates, ranging from 29% to 56% [43–46]. Previous reports have clearly demonstrated that the elevated risk for anal HPV in MSM is increased by HIV infection. Thus, anal HR-HPV infection was up to 4–10 times more frequent in MSM living in many countries outside Africa than in heterosexual men [47, 48]. Finally, our observations confirm that anal HPV constitutes a major infectious health concern in the MSM living in Bangui, highly escalated by HIV infections, and that each MSM community is characterized by local epidemiological specificities rendering necessary their research before intervention.

In our series, the distribution of anal HPV in anal samples appeared quite atypical, with HPV-35 being the predominant genotype. Furthermore, HPV-16 and HPV-18 were very poorly represented in the study population with the most prevalent 9-valent vaccine HR-HPV being HPV-58. This unusual distribution mirrors the previous observations by Nowak and colleagues, reporting that anal samples from MSM living in Nigeria harbored HPV-35 as the predominant genotype, HPV-16 and HPV-18 as minor genotypes, and HPV-58 as the most prevalent 9-valent vaccine genotype [7]. Interestingly, MSM infected by HPV-35 in our series and in MSM living in Nigeria [7], were all co-infected with HIV likely suggesting that HIV infection may play a role in the persistence of this unusual HPV type as a predominant genotype. Likewise, another recent study conducted in the United States of American highlighted the low proportion of HPV-16 and HPV-18 in anal samples from young black American MSM [42]. In contrast, anal HPV-16 and HPV-18 were the most prevalent in MSM living in South Africa, including a majority (67%) of mixed race/colored and white people and only (31%) one-third of black individuals [6]. Taken together, these observations suggest the possibility of a regional distribution in molecular epidemiology of HR-HPV within the diverse MSM communities inside the sub-Saharan African continent [49]. Thus, it is possible to hypothesize that anal cancers in certain black African MSM populations may be due to other HR-HPV rather than HPV-16 and HPV-18, which constitute the HR-HPV types involved in more than 89% of all anal cancers in MSM living in Western countries [50, 51]. Interestingly, others studies on HPV infection in women living in Nigeria have also reported HPV-35 as the predominantly isolated genotype [52,53]. In the Central African Republic, there is no data on the HPV type specific prevalence in the general population. It would be interesting to check whether the unusual HPV genotype distribution found in our Central African MSM series is similar to that in the female or general population. Further studies are nevertheless needed to determine the natural history and the burden of HPV-associated diseases in black African MSM in order to confirm our observations and to formulate effective and adapted HPV vaccine strategies towards young African MSM.

HPV types included in the prophylactic Gardasil-9^®^ vaccine were detected in the majority of HPV-positive anal samples. Around 70% of all anal HPV-positive individuals harbored at least 1 of the 9-valent HPV vaccine genotypes and 45% of HPV-positive anal specimens contained multiple HPV vaccine types. High rates of 9-valent HPV vaccine types in anal canal of MSM were previously reported in South Africa (57%) [6].These observations indicate that MSM living in Bangui, as other MSM populations, constitute a key target population for HPV vaccination with the current prophylactic Gardasil-9^®^ vaccine, which would potentially prevent most of HPV infections and associated anal diseases. However, anal HR-HPV not included in the prophylactic nonavalent vaccine, including the unusual HPV-35, were found in one-third of the study's MSM, indicating that the current HPV vaccine may be insufficient to prevent HPV-related diseases in a significant proportion of the MSM community living in Bangui. Thus, the guidelines on HPV immunization recommended in 2015 by the American Cancer Society (ACS), which integrate HPV vaccination up to 26 years for young MSM with the current two large spectrum HPV vaccines [54, 55], because HPV-16 and HPV-18 are mostly involved in HPV-associated anal cancer in Western countries [50, 51], may be poorly adapted to the MSM community living in the Central African Republic and other sub-Saharan African settings.

*In vitro* interactions between HPV and HSV-2 [16], in the context of high HSV-2 prevalence in sub-Saharan Africa [18], prompted us to evaluate the possible association between anal HSV-2 DNA shedding and anal HPV detection. The rate of anal HSV-2 DNA detection in our series (11.9%) was similar to that (10.6%) reported in a recent meta-analysis on MSM living in China [56]. However, no association between anal shedding of HSV-2 and HPV detection could be found.

Multiple HR-HPV was frequently detected in anal swabs from Bangui’s MSM, mainly in HIV-infected individuals. Multiple HR-HPV in MSM is yet to be well documented [6, 21, 42, 51,57]. High rates (91–94%) of multiple anal HPV infections with numerous different HPV genotypes ranging from 0 to 18 (mean, 4.8–5.0) were reported in HIV-positive MSM living outside Africa, such as North Canada and Thailand [58, 59]. In the present series, multiple HR-HPV infections were more frequently detected in HIV-infected than HIV-negative MSM and multiple anal HR-HPV infection was 15-times more frequent in HIV-positive than in HIV-negative MSM (univariate analysis). Indeed, high-risk sexual behavior, including exclusive sex with other men while being HIV-infected, constitutes a significant cofactor strongly associated with increased risk of multiple anal infections with HR-HPV genotypes [13–15]. In our study MSM population, MSM-exclusively showed 7-times more frequently receptive than insertive anal intercourse (88% *versus* 13%). Furthermore, MSM-exclusively were all HIV-positive (100%), thus more frequently infected by HIV than those having sex with both men and women (62%).

The principal negative modulatory cofactor for HPV anal carriage was behavioral, the MSM practicing condomless receptive anal intercourse being at more risk to be infected with HPV than those practicing insertive anal intercourse. Condomless receptive anal intercourse in MSM is well known to constitute the main sexual risk factor associated with HPV infection [6, 14, 15].

These findings are reminiscent to previous reports on HIV transmission demonstrating that MSM practicing receptive anal intercourse are at higher risk for HIV acquisition than their insertive partner [60, 61]. More generally, a receptive partner is more vulnerable to HPV infection than insertive partner in anal intercourse, as previously demonstrated in a recent study on 733 HIV-infected individuals (538 MSM, 195 heterosexual) that showed the prevalence, clearance and incidence of HPV infection was higher in the anal mucosa (73%, 30% and 36%, respectively) than in penile mucosa (26%, 56% and 17%, respectively) [62]. Indeed, the rectal mucosa, which consists of a single layer of epithelial cells, may break easily, exposing the epidermoid cells of the basal lamina, thus facilitating their infection by HPV [63]. Furthermore, the anal mucosa is relatively large and the rectal receptacle may retain pathogens [63, 64]. Circumcised men, as MSM living in Bangui, have keratinized external mucosa of the penis that provides strong epithelial barrier hampering HPV infection [63, 64].

Finally, HIV-positive MSM reported practicing condomless receptive oral sex were more frequent than HIV-negative MSM, and this sexual practice constituted a risk factor was strongly associated with having anal infection with any type of HPV and particularly HR-HPV. Hu and colleagues have previously reported in HIV-infected Chinese MSM that oral sex, even protected, is closely linked with anal HPV infection, suggesting that other high-risk practices of the sexual repertoire, yet unevaluated, may be associated [44]. Taken together, these observations are similar to a previous report in South Africa where the population of HIV-positive MSM-exclusively constituted a high-risk group accumulating several risky sexual behaviors and multiple anal HR-HPV infections [6]. In short, these observations emphasize the urgent need to implement adapted intervention strategies towards African MSM to reduce sexual risk behaviors. Intervention strategies such as counseling for HIV and sexual risk behaviors have been shown to reduce the incidence of HIV and STI among MSM [65–68]. Furthermore, scaling up prevention strategies against HR-HPV infections and associated cancers adapted for this at-risk vulnerable population should be prioritized. Targeted behavioral interventions may include promotion of consistent condom use [65–68], clinical examination with digital ano-rectal touching (“DARE”) which is an important tool for the early detection of precancerous lesions and anal cancer [69], medical male circumcision to reduce HIV incidence, as well as prevalence and persistence of anal HR-HPV infections [70], and finally HPV prophylactic vaccination of young MSM [55].

In conclusion, MSM community living in Bangui constitutes a very high risk population for both HIV infection and HR-HPV anal infections, and should urgently receive adapted STI and anal cancer screening and care.

Our study had some limitations. Indeed, the recruitment of participants from only the CNRIST/TAR of Bangui as well as the small sample size of our study population, may have introduced selection and information bias. Thus, the study participants may be not completely representative of the MSM community of the Central African Republic, especially regarding prevalences of HIV and anal HPV, and the genotypes distribution of anal HPV. Furthermore, some risk factors may have been underestimated in the statistical analyses.

## Supporting information

S1 Data Excel data sheet containing raw data on socio-demographic, behavioral characteristics, as well as the results of different biological analyzes carried out on MSM living in Bangui included in the study. (XLSX)
